# Microwave-Assisted Syntheses of 1-Acetyl 2-Methylbenzimidazole Sodium Bisulfate pH-Responsive Ionic Draw Solute for Forward Osmosis Applications

**DOI:** 10.3390/membranes15110325

**Published:** 2025-10-26

**Authors:** Ahmed A. Bhran, Abdelrahman G. Gadallah, Hanaa M. Ali, Sahar S. Ali, Hanaa Gadallah, Rania Sabry

**Affiliations:** 1 Chemical Engineering Department, College of Engineering, Imam Mohammad Ibn Saud Islamic University (IMSIU), Riyadh 11432, Saudi Arabia; agadallah@imamu.edu.sa; 2 Chemical Engineering Department, Engineering Research Institute, National Research Centre, Cairo 11241, Egypt; hanaamohamedaly2007@gmail.com (H.M.A.); sahar_saad_ali@yahoo.com (S.S.A.); hanaagadallah@hotmail.com (H.G.); rania_nrc@hotmail.com (R.S.)

**Keywords:** forward osmosis, membrane desalination, draw agents, 2-methylbenzimidazole derivatives, brackish water

## Abstract

This work is related to the development of a highly efficient pH-responsive ionic draw solute for forward osmosis applications utilizing microwave-assisted fast heating. This solute is classified as an ionic compound, a sodium salt originating from imidazole, with the scientific acronym 1-acetyl-2-methylbenzimidazole sodium bisulfate (AMBIM-Na). The synthesized compound was analyzed by X-ray diffraction (XRD), Fourier transform infrared spectroscopy (FTIR), as well as additional physical characteristics. The baseline performance was initially evaluated at various molar concentrations against distilled water as the feed solution (FS). The results indicated that the produced solute exhibits elevated osmotic pressure, resulting in a water flux of up to 130 LMH for a 1 M concentration, coupled with the absence of reverse salt flux. The synthesized AMBIM-Na at a concentration of 1 M was utilized as a draw solution (DS) against synthetic brackish water. The water flux declined progressively with the increase in FS concentration, decreasing from 130 LMH with distilled water to 99, 70, and 41 LMH at NaCl concentrations of 5, 10, and 15 g/L, respectively. The regeneration of the draw solute was assessed using pH adjustment, revealing that 100% regeneration occurs by reducing the pH to 2.

## 1. Introduction

Recently, due to rapid population increase, accelerated industrialization, and the depletion of accessible freshwater supplies, shortages of water are emerging as a significant global concern [1]. According to Earth.Org, as of March 2024, around 703 million individuals lack access to water, and over 2 billion do not have access to clean drinking water services. Moreover, under the probable climate change scenario, by 2030, approximately fifty percent of the global population will reside in regions suffering significant water stress [2]. Several approaches have been proposed for dealing with the water scarcity situation by identifying new water resources, planning for urban scarcity, and developing climate-resilient water sources, including the use of deeper groundwater reserves through solar-powered water networks. Also advancing water storage through small-scale retention structures, managed aquifer recharge (where water is pumped into underground reserves to improve its quality), and rainwater harvesting. Improving the efficiency of water resources includes desalination and water reuse, which are recognized as effective methods for rapid supply without harming natural freshwater ecosystems [1]. Desalination is a process employed to eliminate salt and contaminants from saline water sources such as brackish water and seawater [3]. Numerous desalination processes have been developed, including vapor compression (VP), multi-stage flash evaporation (MSF), multi-effect distillation (MED), electrodialysis/electrodialysis reversal (ED/EDR), reverse osmosis (RO), nanofiltration (NF), forward osmosis (FO), and others [4]. The main negative aspects of saltwater desalination involve the significant energy consumption of its procedures, the elevated costs of relevant equipment, and the accompanying environmental issues. The substantial capital expenditure (CAPEX) and operational expenditure (OPEX) related to saltwater desalination often result in costs that are ultimately passed on to end consumers in the price of desalinated water [5].

Since the early 2000s, FO has garnered considerable interest as a greener membrane technology for desalination and water treatment, offering advantages of reduced energy expenditure and decreased membrane fouling relative to conventional membrane processes such as RO and NF [6]. The FO process employs a semi-permeable membrane and two solutions: an FS and a DS, each of which has a differing osmotic pressure (OP). The OP disparity serves as the impetus for FO processes, promoting water transfer from the FS (low OP side) to the DS (high OP side), while the semi-permeable membrane efficiently obstructs ion migration [1,7,8]. The high hydraulic pressure in the FO process, as opposed to RO, could reduce the usage of energy in electrical pumping. Additionally, the reduction of fouling propensity and fouling reversibility improvement could extend the membrane’s lifespan and decrease the whole operational costs [9,10,11]. The difference in OP across the FO membrane serves as the process’s driving power; hence, selecting an appropriate draw agent requires considering variables like chemical stability and matching with the FO membrane [12]. The draw solute must effectively diminish the chemical potential of water, thereby yielding a large OP in the FO process. Meanwhile, the draw solute is anticipated to be simply removable from the diluted DS in the subsequent process, enabling the recovery of the draw solute for reuse and the extraction of pure water [13]. In recent years, various material classes, including organic and inorganic solutes, have been investigated and assessed as possible optimal draw solutes [14]. Inorganic salts, particularly sodium chloride (NaCl), are extensively utilized owing to their low price and comparatively high OP. A 0.5 M NaCl DS exhibited a water flux of 20.5 LMH, accompanied by a significant reverse solute flux of 5.9 g/m^2^h [15]. Multivalent salts (CaCl_2_, MgCl_2_) have been examined as draw solutes to mitigate reverse solute flux, attributed to their larger hydrated radius and better electrostatic repulsion; they exhibited water fluxes of 8.36 and 9.74 LMH at a concentration of 0.5 M, with associated reverse solute fluxes of 3.34 and 3.89 g/m^2^h, respectively. But it has been shown that Mg^2+^ and Ca^2+^-based desalination systems pose a significant danger of membrane scaling [16]. In addition, the recoveries of these draw solutes with RO or NF membranes present an obstacle due to their substantial energy consumption during the regeneration process [14]. To limit reverse solute flux, organic draw solutions with macromolecular structures, including polyelectrolytes, polymers, and nonpolar polymers, have been implemented in saltwater desalination and wastewater treatment [17].

The recovery of inorganic or organic DS is energy-intensive and expensive, which conflicts with the objective of FO. Lately, many types of draw solutes employing distinct regeneration methods have been documented, including direct usage, thermal and membrane separation, chemical sedimentation, and stimuli-responsive techniques. Responsive draw solutes are a category of intelligent solutes capable of substantially decreasing the OP of diluted DSs when subjected to an exterior stimulus, such as temperature, magnetic or electric field, pH, or light [13]. The implementation of responsive materials as draw solutes would streamline the recovery process through basic stimuli. Lately, responsive draw solutes have been investigated for salty water desalination, heavy metal removal, and wastewater treatment [18,19]. The pH-responsive DS switches its solubility in the solvent pursuant to the pH level of the DS. Generally, all pH-responsive DSs exhibit elevated OP and a robust capacity to extract water in normal or basic conditions. The pH-responsive draw solutes precipitate as particles or undergo polymerization in acidic conditions [20]. The pH-sensitive properties of pH-responsive draw salts arise from their negatively charged groups or ions, whose structure, ionization state, or hydrodynamic diameter alters upon interaction with hydrogen ions. For instance, (1-(3-aminopropyl)-imidazole) propane-sulfonate, the speciation of ethylene diamine tetra acetic acid disodium salt, the acrylic acid component of polyacrylic acid sodium salts, and molybdenum acid ions of (NH_4_)_6_Mo_7_O_24_ are most frequently used as pH-responsive draw solutes [21,22]. Studies have shown that pH-responsive DSs exhibit greater OP and water flux compared to equivalent concentrations of NaCl DSs. This may be ascribed to the pH-responsive draw solutes’ capacity to interact with water molecules via hydrogen bonds, thereby forming a polymer net [23]. The pH-responsive DS exhibits a minimal reverse salt flux owing to its substantial diameter, which surpasses the membrane pore size, and its elevated charge density, which yields electrostatic repulsion with the membrane surface [24,25].

2-Methylimidazole is a cost-effective commercial substance, possessing structural similarities to imidazole. The ortho-position of the five-membered ring is exchanged with a methyl group, resulting in a fundamental difference. 2-Methylimidazole is less acidic than imidazole; however, it retains numerous biological and chemical-related functions. The imidazole protonation or deprotonation alters the acidity and basicity effects, exemplified by the histidine in enzymes [26]. Yen et al. synthesized 2-methylimidazole-based organic compounds and evaluated their efficacy as draw solutes [27]. They showed that these compounds exhibit favorable solubility through hydrogen bonding and charge ionization of the salt in water, with moderate molecular weights ranging from 82 to 500. The substitution of the ortho-position with a methyl group prevents deprotonation (in contrast to the imidazolium salt), and the nitrogen atom in the five-membered ring is readily accessible for alkylation reactions. Liu et al. developed pH-responsive sodium salts of an imidazole-based ionic solid (im-IS), specifically im-IS-Na and im-IS-2Na, to serve as draw solutes for FO applications [28]. It proved that both synthesized compounds possess an extended structure and include several ionic moieties. These characteristics enable them to produce an adequate OP for FO separation and almost zero reverse solute flux. The findings revealed that im-IS-Na and im-IS-2Na at 1.0 M yield water fluxes of 27.5 LMH and 32.0 LMH, respectively, reflecting an increase of up to 128% compared to traditional draw solutes. Furthermore, im-IS-Na and im-IS-2Na may be efficiently recovered through pH adjustment, reaching a yield of 98%.

The preparation of 2-methylimidazole derivatives typically involves stirring ingredients at a temperature of 95 °C for 2 h [27,28]. Microwave irradiation affords a substitute for traditional heating methods. It employs the capability of mobile electric charges in liquids or conducting ions in solids to convert electromagnetic energy into heat [29]. The microwave-assisted synthesis is environmentally friendly and delivers superior yields [30,31]. Microwave synthesis is significant due to its quick reaction times, broad variety of reactions, minimal exposure to hazardous compounds, and optimal energy utilization [32,33]. Microwave-assisted synthesis is predominantly utilized in both industrial applications and academic studies [34]. Recently, the benefits of microwaves have made it possible to synthesize in household microwave ovens, serving as a straightforward, rapid, and cost-effective instrument, operating on the same premise as an advanced synthesis oven. The primary distinction is that household microwave ovens only control irradiation time while not providing accurate pressure and temperature regulation during synthesis; still, commendable outcomes have been achieved [35,36].

This study seeks to create a highly effective pH-responsive ionic draw solute produced from imidazole by microwave-assisted synthesis for FO applications. The scientific name of the draw solute is 1-acetyl-2-methylbenzimidazole sodium bisulfate (AMBIM-Na). The obtained draw solute was characterized using XRD, FTIR, and other physical characteristics. The efficacy of the draw solute at varying molar concentrations was initially examined with pure water as FS. The draw solute was assessed for desalination effectiveness with synthetic brackish water. Furthermore, the regeneration of the draw solute was ultimately assessed using pH regulation. To the best of our knowledge, this investigation represents the first time use of this compound in the FO system.

## 2. Materials and Methods

### 2.1. Materials

#### 2.1.1. Chemicals

All chemicals employed are of analytical grade and utilized without additional purification. The components include Orthophenylenediamine, sulfuric acid (H_2_SO_4_, 98%), anhydrous acetic acid, sodium hydroxide (NaOH, 98%), and sodium chloride (NaCl). The chemicals were picked up from Sigma-Aldrich Chemical Company (St. Louis, MO, USA)

#### 2.1.2. Membranes

The cellulosic woven FO membrane from HTI Company (Albany, OR, USA) was used with a 50 μm thickness. It is relatively hydrophilic and smooth [37]. Coupons of virgin membrane were sized and soaked in distilled water overnight before each experiment.

### 2.2. Methods

#### 2.2.1. Preparation of the pH-Sensitive DS

Orthophenylenediamine is mixed with anhydrous acetic acid in a 1:2 ratio and then drops of concentrated sulfuric acid are added to remove water. The mixture is heated rapidly in a household microwave oven (Jac company, model: NGM-2525; Seoul, Pepublic of Korea) at 200 watts for 30 s. The reaction mixture was cooled till crystals of AMBIM-HSO4 were formed and then dried at ambient temperature for characterization. Finally, the resultant crystals are neutralized with 1 M sodium hydroxide, resulting in the formation of AMBIM-Na, which is used as a draw solution for the FO system.

#### 2.2.2. Characterizations of AMBIM-HSO_4_

The chemical structure of AMBIM-HSO_4_ was identified using FTIR, which was measured at a spectrum range of 4000 to 400 cm^−1^. The test was carried out by JASCO FTIR-6100 spectrophotometer delivered from JASCO company (Tokyo, Japan). XRD examination was conducted using an XRD-6000 apparatus (Shimadzu company, Kyoto, Japan) with CuKα (1.54060 Å) radiation, working at 30 mA and 45 kV, scanning at 2° min^−1^ (0.02° steps).

#### 2.2.3. Determination of Osmotic Pressure, π

OP of AMBIM-Na DSs at varying molarities (0.25–1 M) was experimentally determined using the freezing point depression method [38], as outlined in our prior research [39,40]. The OP (π) of the DS is estimated using Equation (1) [38].

(1) $$\pi =\frac{\Delta T}{1.86}\times 22.66\;(bar)$$

where ΔT is the difference between the deionized water freezing point and that of the DS.

#### 2.2.4. Draw Solute Performance

**Figure 1:**
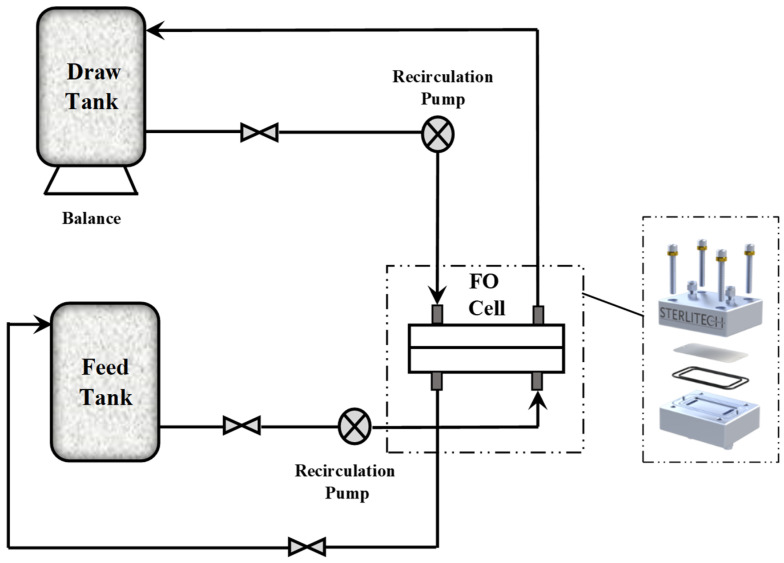
Bench-scale forward osmosis experimental setup.

The FO performance of the prepared DS at various concentrations was assessed by a bench-scale FO cell (CFO42) delivered from Sterlitech company (Auburn, (WA), USA). The device depicted in Figure 1 comprises a single membrane unit split into two similar channels; the external dimensions of the cell are 12.7 × 10 × 8.3 cm^3^, while the active area is 9.2 × 4.6 cm^2^. The membrane’s effective surface area measures 42 cm^2^.

The flow of the two solutions (draw and feed) was engineered to be counter-current on both sides of the membrane. The solution flow was regulated independently by a gear pump model 81808 (Cole-Parmer company, Vernon Hills, (IL), USA) at a rate of 0.857 L min^−1^. The DS tank was positioned on an analytical balance model XP8002S (Mettler Toledo company, Columbus, (OH), USA) to record the variation in DS weight. A Myron L Ultrameter conductivity meter (Carlsbad, (CA), USA) was used to quantify the total dissolved solids of the DS.

The arrangement of the membrane was specified such that the FS contacted the active layer [Al-FS] (Cath et al., 2006) [41]. The performance was assessed in 1 h by measuring the permeate flux (Jw) and reverse salt flux (Js).

The FS employed in these studies was distilled water. A stabilization period of ten minutes was selected, after which the permeate flux was determined by measuring the change in DS weight with time using Equation (2) [42]

(2) $$Jw=\frac{∆V}{A\;∆T}$$

where Jw is water flux (LMH), ΔV is the permeate volume (L) that passes through the membrane during the time interval Δt (h), and A (m^2^) is the effective membrane area. The reverse salt flux (Js, g/m^2^ h) was determined by Equation (3) [43]

(3) $$Js=\frac{\left(C_{f}V_{f}-C_{o}V_{o}\right)}{A\;∆T}$$

where C_f_ and C_0_ (mol/L) are the final and initial salt concentration of the FS, and V_f_ and V_0_ (L) are the final and initial volume of the FS.

#### 2.2.5. Application for Brackish Water Desalination

The efficacy of the synthesized AMBIM-Na at the optimal concentration in FO applications using varying concentrations of brackish water as feed was examined. The FS comprised synthetic brackish water, formulated with a commercial NaCl solution at doses of 5, 10, and 15 mg/L. The performance was appraised based on water fluxes through the membrane.

#### 2.2.6. DS Regeneration

The AMBIM-Na solution was demonstrated to be entirely soluble under neutral conditions (pH range from 6 to 7). Gradual precipitation occurred by acidifying the solution to pH 2 with drops of 1 M H_2_SO_4_. The precipitate was subsequently filtered, washed, and dried to obtain the recycled crystals.

## 3. Results and Discussions

It should be noted that the obtained experimental results were triplicate experiments, and each measure corresponds to the average value. The obtained standard deviation (SD) for these replicates is lower than 2%, which indicates the reliability and validity of these data.

### 3.1. Syntheses and Characterizations of AMBIM-HSO_4_

**Figure 2:**
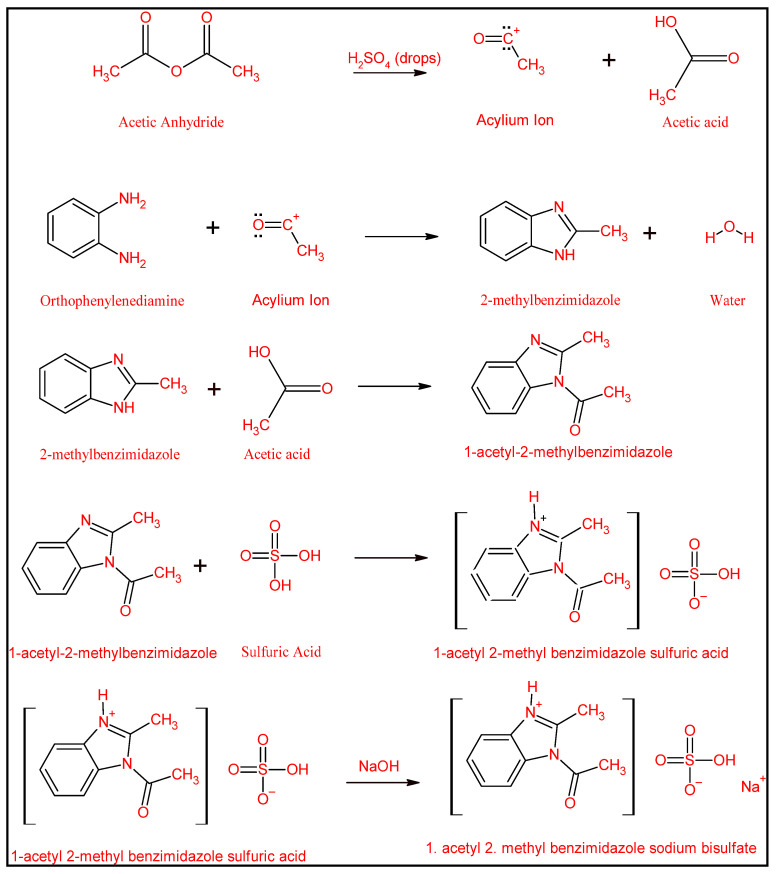
Reaction mechanism for AMBIM-Na syntheses.

Figure 2 elucidates the proposed reaction pathway for the synthesized 2-methylbenzimidazole derivative, in which acetic acid, in the presence of sulfuric acid, generates a stable intermediate known as the acylium ion. This ion has greater stability in strong mineral acids such as sulfuric acid. The generated acylium ion can be attacked by the diamine from o-phenylenediamine. The electron-dense nitrogen atom attacks the carbonyl carbon of the acylium ion. Subsequently, the liberation of water facilitates cyclization. The presence of acetic acid can react with the 1 position, resulting in the formation of 1-acetyl-2-methylbenzimidazole. Additionally, 1-acetyl-2-methylbenzimidazole reacts with sulfuric acid to yield 1-acetyl-2-methylbenzimidazole sulfuric acid (AMBIM-HSO_4_). Ultimately, to utilize the synthesized substance as a draw solute, it is neutralized with sodium hydroxide to enhance its solubility in water, resulting in the synthesis of 1-acetyl-2-methylbenzimidazole sodium bisulfate (AMBIM-Na).

**Figure 3:**
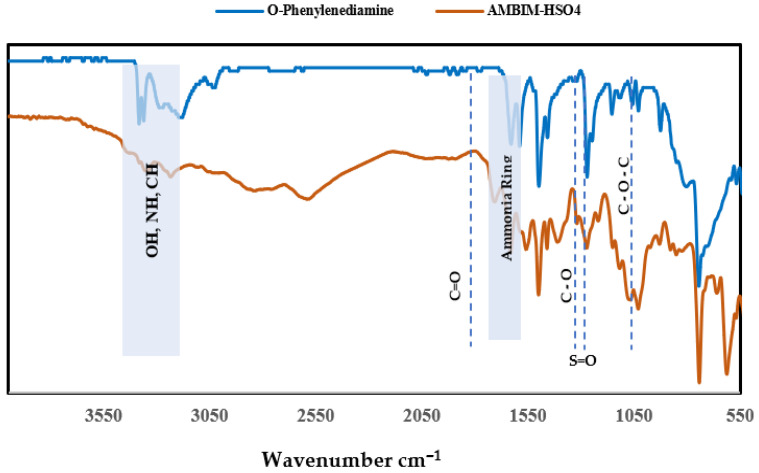
FTIR spectra of AMBIM-HSO_4_ and orthophenylenediamine.

The mechanism is validated by comparing the FTIR spectra of the produced compound with that of orthophenylenediamine (Figure 3), which exhibits two peaks at 3384 and 3363 cm^−1^, indicative of moisture content or the presence of trace contaminants. The peaks detected at 3400–3000 cm^−1^ result from the overlapping peaks of OH from sulfuric acid and NH and CH stretching of the imidazole ring. The region is delineated into three distinct segments: firstly, a pronounced peak at 3282 cm^−1^ attributed to isolated non-hydrogen-bonded N–H groups; secondly, a broad asymmetric peak centered approximately at 3187 cm^−1^ resulting from self-associated hydrogen bonding of CH stretching within the benzimidazole ring (N–H group); and thirdly, a very low-intensity peak at 3029 cm^−1^ corresponding to the stretching modes of aromatic C–H groups. The C=C aromatic stretch is observed within the region of 1450–1600 cm^−1^. Benzene rings frequently exhibit ring breathing at approximately 1000 cm^−1^. The out-of-plane bending of imidazole –CH, overlapping with the benzene aromatic skeletal absorption, is seen around 742 cm^−1^. The CH stretching of methyl and aromatic groups occurs around 2925 cm^−1^, while the C–N stretching and in-plane vibration of the imidazole ring are observed about 1270 cm^−1^.

The combined vibrations of C=C and C=N rings are seen at 1589 and 1630 cm^−1^. One unique absorption band is located at 611 cm^−1^, corresponding to the deformation vibration of aromatic CH in the meta (1, 3) substituted benzene ring. Bands specifically associated with this molecule are observed at 1560 and 1630 cm^−1^, corresponding to the meta-substituted aromatic ring, so verifying the synthesis of methylimidazole. The presence of peaks at 1150 cm^−1^ and 1272 cm^−1^ is attributed to the asymmetric and symmetric vibrations of S=O. Furthermore, the peak at 622 cm^−1^ is attributed to the S-O bond of HSO_3_, while the peak at 1066 cm^−1^ corresponds to C-O-C [44,45]. The C=O stretch typically happens at 1700 cm^−1^, while the C-O stretch in esters containing an acetyl group is observed at 1300 cm^−1^.

**Figure 4:**
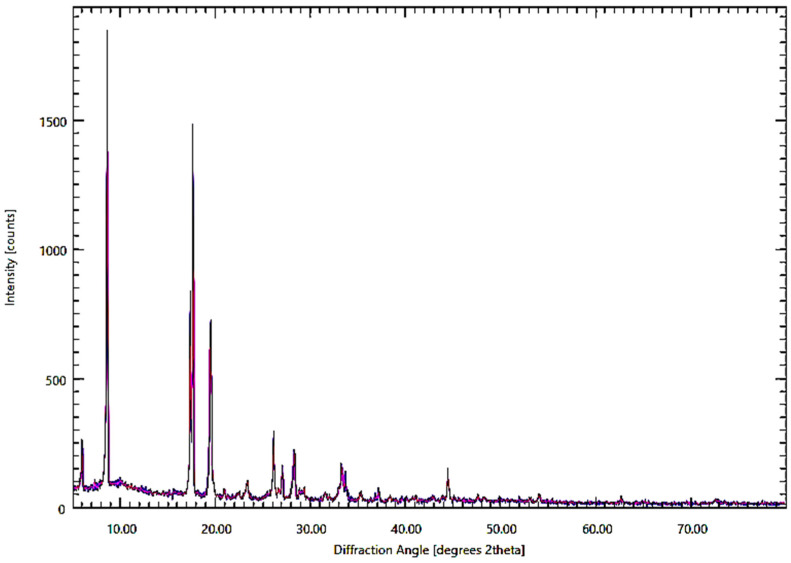
X-ray diffraction (XRD) of AMBIM-HSO_4_ crystals.

Figure 4 elucidates the X-ray diffraction (XRD) pattern of AMBIM-HSO_4_, revealing that the compound is highly crystalline, as evidenced by the presence of sharp, identifiable peaks over a broad range of 2θ. The detection of a prominent, sharp peak at 2θ = 8.66 implies a single crystalline phase, possibly signifying phase purity and confirming the creation of the ionic liquid compound.

The existence of a low-angle peak at 8.66 (d = 10.21 Å) indicates a layered or planar stacking configuration. Furthermore, the pronounced peaks at 2θ = 18 and 19 signify the interaction of the 2-methylimidazole molecule via the π-π link. Additionally, there are two peaks at 25.7 and 29.7 degrees of 2θ that align with HSO_3_.

### 3.2. AMBIM-Na OP

**Figure 5:**
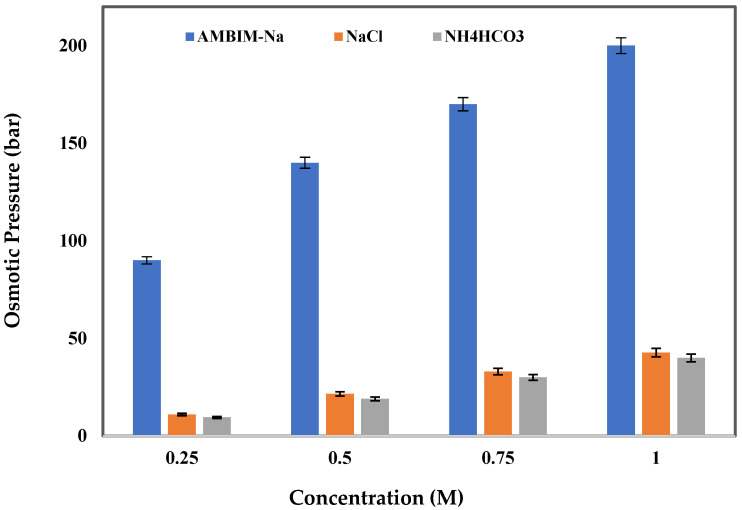
Osmotic pressure of AMBIM-Na, NaCl, and NH_4_HCO_3_ draw solutes at different concentrations.

The OP of synthesized AMBIM-Na solutions at various concentrations was determined and compared with that of sodium chloride (NaCl) and ammonium bicarbonate (NH_4_HCO_3_) solutions, which are established as traditional draw solutes. Figure 5 displays the OP of the three draw solute solutions under identical conditions. The synthesized compound exhibited significantly greater OP compared to traditional NaCl and NH_4_HCO_3_ draw solutes, with a maximum examined concentration of 1 M yielding 200 bars of OP for AMBIM-Na, whereas NaCl and NH_4_HCO_3_ generated only 42.7 and 40 bars, respectively.

The chemical composition of the synthesized compound (Figure 2) signifies that its elevated OP can be ascribed to the formation of hydrogen bonds between the nitrogen of the imidazole ring and the oxygen of the acetyl group with water, along with the presence of a sulfonic group that exhibits high hydrophilicity towards water [27]. Liu et al. pointed out that imidazole-based ionic solutes, featuring larger configurations and more complex chemical compositions, exhibited higher OP compared to NH_4_HCO_3_ and NaCl [28]. Moreover, as observed by Wu et al., a multi-charged electrolyte generates elevated OP due to the release of greater amounts of ionic particles in solution [23].

### 3.3. FO Performance

**Figure 6:**
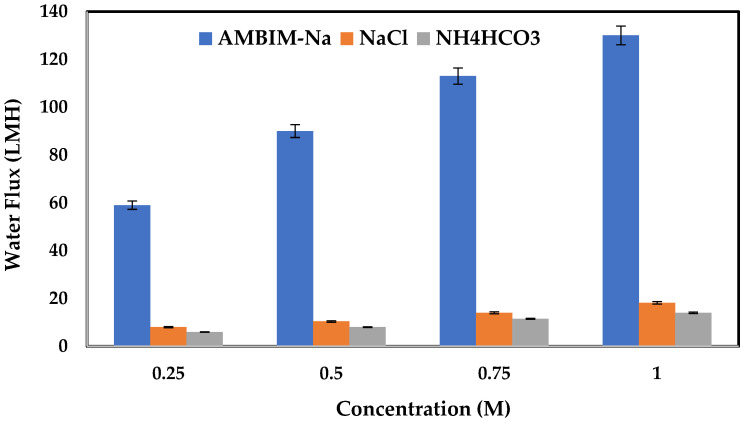
Water flux for FO AMBIM-Na, NaCl, and NH_4_HCO_3_ draw solutes at different concentrations and distilled water as feed solution (Al-FS).

**Figure 7:**
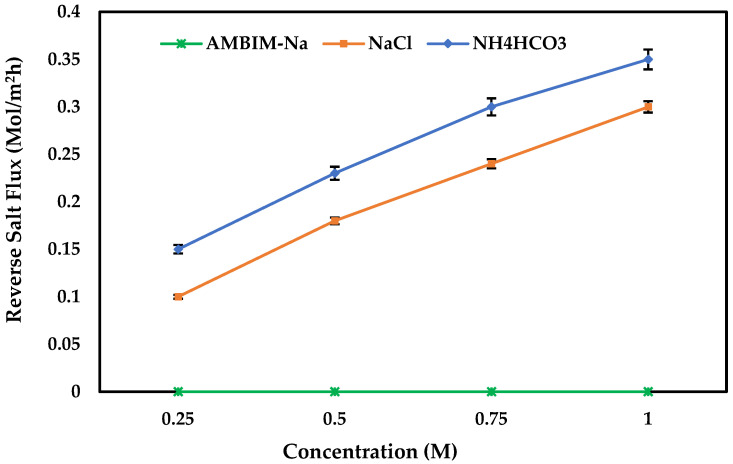
Reverse salt flux for FO operation with AMBIM-Na, NaCl, and NH_4_HCO_3_ draw solutes at different concentrations and distilled water as feed solution (Al-FS).

Figure 6 shows the effect of AMBIM-Na concentration as draw solutes on water flux through the FO membrane by using distilled water as a feed solution compared with NaCl and NH_4_HCO_3_ draw solutes at the same conditions. The concentrations were varied between 0.25 and 1 M. As observed in OP results, it is clear that the performance of AMBIM-Na is greatly higher than that obtained with NaCl and NH_4_HCO_3_ to draw solutes. In addition, high flux values were obtained at higher concentrations, then they decreased gradually by decreasing the molarity, in which for 1 M concentration the flux reached 130, 18.2, and 14 LMH for AMBIM-Na, NaCl, and NH_4_HCO_3_, respectively, then it decreased to 113, 14, and 11.5; 90, 10.4, and 8; and 59, 8, and 6 at concentrations of 0.75, 0.5, and 0.25 M. On the other hand, no reverse solute flux was detected for all AMBIM-Na concentrations, while NaCl and NH_4_HCO_3_ gave reverse solute flux up to 0.3 and 0.35 mol/m^2^h at 1 M concentration for NaCl and NH_4_HCO_3_, respectively (Figure 7).

The improvement of water flux with rising draw solution concentration is well-documented in the literature. Arkhangelsky et al. explained that water flux did not increase linearly with the concentration of the draw solution [46]. Comparable observations have been reported earlier, and the internal concentration polarization (ICP) effect was proposed as the cause of this behavior [47,48,49]. The impact of ICP on water flux worsened at elevated DS concentrations due to the accumulation of salt ions in the membrane support layer adjacent to the DS. The absence of reverse salt flux can be ascribed to the higher molecular weight of imidazole derivatives and the formation of charged molecules. This matches the findings of Yen et al. [27], who investigated draw solutes utilizing 2-methylimidazole-based compounds in forward osmosis. They concluded that the cation of the charged molecules is significantly more easily held onto the draw solution side, thereby restricting the diffusion of the counter ion.

### 3.4. AMBIM-Na Application

**Figure 8:**
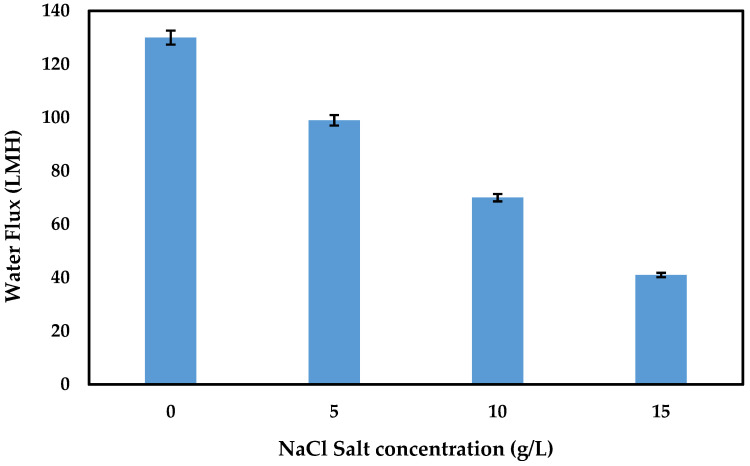
Effect of NaCl feed solution concentration on FO water flux by using AMBIM-Na as DS (Al-FS).

AMBIM-Na at a concentration of 1 M was applied as a draw solution against synthetic brackish water as a feed solution. Figure 8 demonstrates the effect of NaCl concentration on FO water flux; it is observed that water flux decreased gradually with the increase of feed concentration, in which it decreased from 130 LMH with distilled water to 99, 70, and 41 LMH at NaCl concentrations of 5, 10, and 15 g/L.

### 3.5. The Long-Term FO Performance

**Figure 9:**
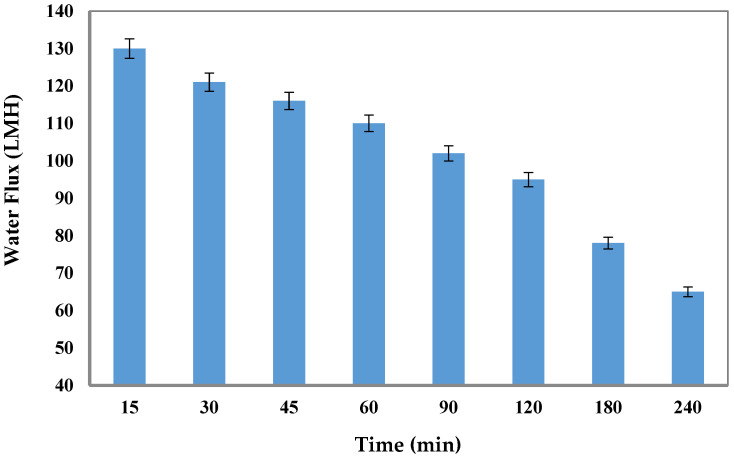
Effect of time on FO water flux by using 1 M AMBIM-Na as DS and distilled water as feed solution (Al-FS).

Figure 9 shows the effect of time on FO performance at 1 M of AMBIM-Na as DS using distilled water as FS within a 240-min permeation time. It was observed that the flux declined from 130 LMH to 65 LMH as a result of the dilution effect.

The results are in good agreement with Cui et al. [50]; they demonstrated the effect of feed solution concentration on FO performance, and they concluded that water flux was decreased with increasing feed concentration due to the decrease in OP difference between the draw and feed solution. In addition, they concluded that increased feed concentration would enhance the diffusion of ions, and the diffusion flux is proportional to the ion concentration difference. However, an increase in feed concentration would increase the feed OP and reduce the overall driving force for water transport. As a result, there is an insignificant reduction in the solute rejection and increase in reverse salt flux.

In general, it is obvious that the performance of the prepared imidazole derivatives as draw solutes in the FO process is significantly higher than the performance of common draw solutes in the literature (see Table 1), except for Wu et al. [23], who achieved results comparable to the present study. The high-water flux values may be attributed to the fact that all pH-responsive draw solutions showed high osmotic pressure due to their ability to drag water molecules through hydrogen bonds and form a polymer network [28]. The above results confirm the formation of new novel solutes that were used for the first time as draw solutes in FO applications.

### 3.6. Regeneration of DS

**Figure 10:**
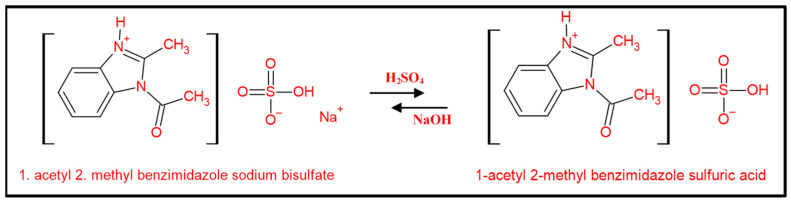
Draw of the solute regeneration/reuse cycle.

To optimize the potency of the produced compound, the draw solute must be recovered and reutilized in the forward osmosis process. The prepared draw solute is sensitive to pH. The alteration of pH will result in the dissolution or recrystallization of the draw solute. As the pH of the solution progressively reduces from neutral to acidic, the solubility of 1-acetyl 2-methyl benzimidazole sodium bisulfate diminishes, resulting in its full precipitation as 1-acetyl 2-methyl benzimidazole sulfuric acid at pH 2 (Figure 10). The draw solute could be neutralized once more with a dilute NaOH solution, converting it into its soluble sodium salt. Additionally, the absence of reverse salt flux (as indicated in Section 3.3) guarantees the weight stability of the draw solute during operation. The regenerated compound was tested for FO performance, and the flux of the FO process was found to be the same. The regeneration/reuse cycle was repeated three times with approximately 99% recovery percent for all regeneration experiments due to some losses through the FO system or during filtration of the solid DS, and water flux values were approximately as demonstrated in Figure 6 [59–130 LMH in the range of DS concentration studied 0.25–1 M]. The results are in good agreement with the literature, as shown in Table 1; the recovery percent ranged between 98 and 100% for all investigated pH-responsive types.

The findings of the regeneration study align well with those of Liu et al., who synthesized pH-responsive sodium salts of an imidazole-based ionic solid as draw solutes for the forward osmosis process [29]. They proved that around 98% of the applied draw solute could be recovered by lowering the pH to 2.

## 4. Conclusions

In this study, a novel pH-responsive ionic draw solute, AMBIM-Na, was successfully synthesized via microwave-assisted methods and evaluated for its performance in FO applications. The physicochemical characterization, including FTIR and XRD analyses, confirmed the formation of a highly crystalline ionic compound with distinctive structural features conducive to high osmotic activity. The synthesized draw solute exhibited significantly higher OP, up to 200 bars at 1 M concentration, compared to conventional draw solutes such as NaCl and NH_4_HCO_3_, which generated 42.7 and 40 bars, respectively. Performance evaluation demonstrated significantly higher water flux (up to 130 LMH) across a wide concentration range with no detectable reverse solute flux, indicating excellent retention properties. When applied to synthetic brackish water, water flux declined predictably with increasing feed salinity, consistent with OP gradient theory. In addition, the compound’s pH sensitivity enabled effective regeneration and reuse through pH-induced solubility switching, with recovery rates near 100% and stable FO performance over multiple cycles. The integration of high OP, excellent water flux, minimal reverse solute loss, and efficient regeneration underscores the potential of this novel imidazole-based compound as a high-performance draw solute for sustainable and cost-effective FO desalination technologies. As far as we are aware, this represents the first application of this compound in FO, demonstrating its potential as a viable and efficient candidate for future innovations in high-performance water purification technologies.

## Figures and Tables

**Table 1:** Comparison between different pH-responsive draw solutes.

“Draw Solute”	Operating Conditions	Feed Solution	Water Flux (LMH)/Reverse Salt Flux (gMH)	pH	% Recovery
This study	1 M	DIW	130/0	2	100
	0.25 M	DIW	59/0		
	1 M	(5–15) g/L NaCL	99–41/0		
Polyoxometalates [24]	(NH_4_)_6_Mo_7_O_24_ (0.4 M)	DIW	16.4/0	<2	100
	Na_6_Mo_7_O_24_ (0.4 M)	DIW	14.2/0		
	(NH_4_)_6_Mo_7_O_24_ (0.4 M)	2 g/L glutathione	10.0/0		
	Na_6_Mo_7_O_24_ (0.4 M)	2 g/L glutathione	9.6/0		
1,4-bis (3-propane-sulphonate sodium)-piperazinediethanesulfonic acid disodium-sulfate [23]	0.24 M	DIW	58.4/0	<7	100
Polyacrylic acid sodium salts (Mw, 2000) [25]	20 wt%	DIW	17.26/0.110	7.78	99.90
(1-(3-aminopropyl)-imidazole) propane-sulfonate (APIS) [22]	1 M	DIW	20–25/0	2	100
EDTA-2Na [51]	0.25 M	DIW	4/0.2	2	98.56
sodium salts of imidazole-based ionic solid, im-IS-Na and im-IS-2Na [28]	1 M im-IS-Na 1 M im-IS-2Na	DIW	32/0 27.5/0	2	98

## Data Availability

The raw data supporting the conclusions of this article will be made available by the authors on request.
